# The gene diagnosis of neurofibromatosis type I with headache as the main symptom: A case report and review of the literature

**DOI:** 10.3389/fneur.2022.874613

**Published:** 2022-08-01

**Authors:** Ming Gao, Haokun Liu, Qiying Sun, Guang Yang

**Affiliations:** ^1^Department of Geriatrics, Xiangya Hospital, Central South University, Changsha, China; ^2^Department of Neurology, Xiangya Hospital, Central South University, Changsha, China; ^3^Department of General Medicine, Xiangya Hospital, Central South University, Changsha, China

**Keywords:** neurofibromatosis type I, headache, gene diagnosis, atypical manifestation, literature review

## Abstract

Neurofibromatosis type I (NF1) is an autosomal dominant disease. Some NF1 patients experience atypical clinical manifestations, genetic testing is not widely available, and the types of mutations vary; thus, they are prone to misdiagnosis and missed diagnosis. Although headache is not included in the diagnostic criteria for NF1, the incidence of headache in NF1 patients is not low. We report an NF1 family in which the proband presented with prominent headache and atypical clinical presentation, with limited skin pigmentation. We identified a frameshift mutation (c.1541_1542del, p. Q514Rfs^*^) in the *NF1* gene by whole-exome sequencing of this family, and the patients were diagnosed with NF1. We hope to attract the attention of clinicians to these patients and improve genetic testing as soon as possible to increase the diagnosis rate.

## Introduction

Neurofibromatosis type I (NF1) is an autosomal dominant condition with a prevalence of ~1/2,500–1/3,500 (1). According to the diagnostic criteria set by the National Institutes of Health, NF1 has two or more of the following characteristics: six or more café au lait patches, two or more neurofibromas, or one plexiform neurofibroma, axillary or inguinal freckling, Lisch nodules, optic glioma, a first-degree relative diagnosed with NF1, or a characteristic osseous lesion (2, 3). However, until genetic testing results emerge, a significant number of patients exhibit atypical manifestations [Such as headache (4), abdominal pain (5, 6), asthenia (7, 8), and shortness of breath (9)]. Although some skin manifestations may occur, they can be easily missed by clinicians. Therefore, physicians must raise awareness regarding NF1 and be vigilant for its atypical clinical symptoms.

Headache is the most common neurological symptom and is not specific. However, the incidence of headache in NF1 patients was not low. In some surveys, the incidence of headache in NF1 patients ranged from 25 to 30%, with an average of one in every three to four patients (10). Here, we report an NF1 family in which the proband presented with headache as the prominent clinical presentation and only some café au lait patches. The laboratory examination results were unremarkable. Head magnetic resonance imaging (MRI) revealed multiple abnormal signal foci in the bilateral basal ganglia, thalamus, and pons. Whole-exome sequencing (WES) and multiplex ligation-dependent probe amplification (MLPA) identified a frameshift mutation (p. Q514Rfs^*^). Headache is not included in the diagnostic criteria for NF1 because it is not specific; however, we hope to improve clinicians' understanding of such patients and conduct genetic testing to confirm the disease as soon as possible.

## Case description

A 13-year-old adolescent boy presented with the chief complaint of recurrent headache for 1 month. One month prior to the consult, the patient developed headache without an obvious trigger, mainly concentrated in the left temporo-occipital region, mild to moderate (2–4 points, total score of 10 points) (11), usually characterized by dull pain, accompanied by vascular pulsation, and occasionally tingling, lasting from several seconds to several minutes, and had four to five attacks in the preceding month. There were no prodromal or accompanying symptoms of aura, photophobia, phonophobia, nausea, or change in level of consciousness. No dizziness or any other neurological symptoms were noted. The patient had no history of hypertension, migraines, sinusitis, or the use of drugs that could cause headaches as an adverse effect. The patient was transferred to our hospital due to headache of unknown cause.

**Figure 1:**
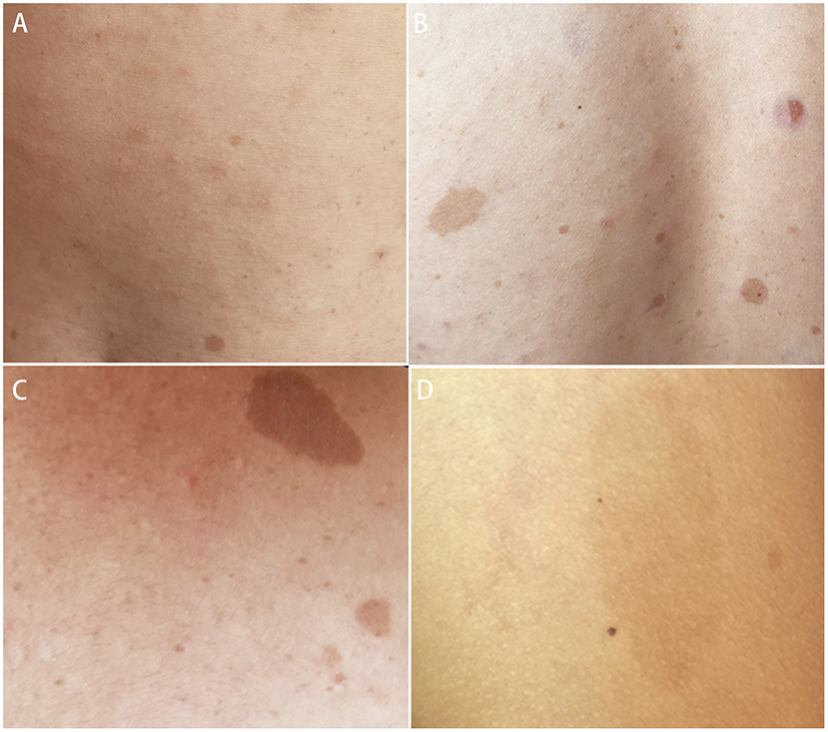
Light brown plaques were seen on the chest and abdomen **(A)**, back **(B)**, shoulder **(C)**, and left upper arm **(D)**.

**Figure 2:**
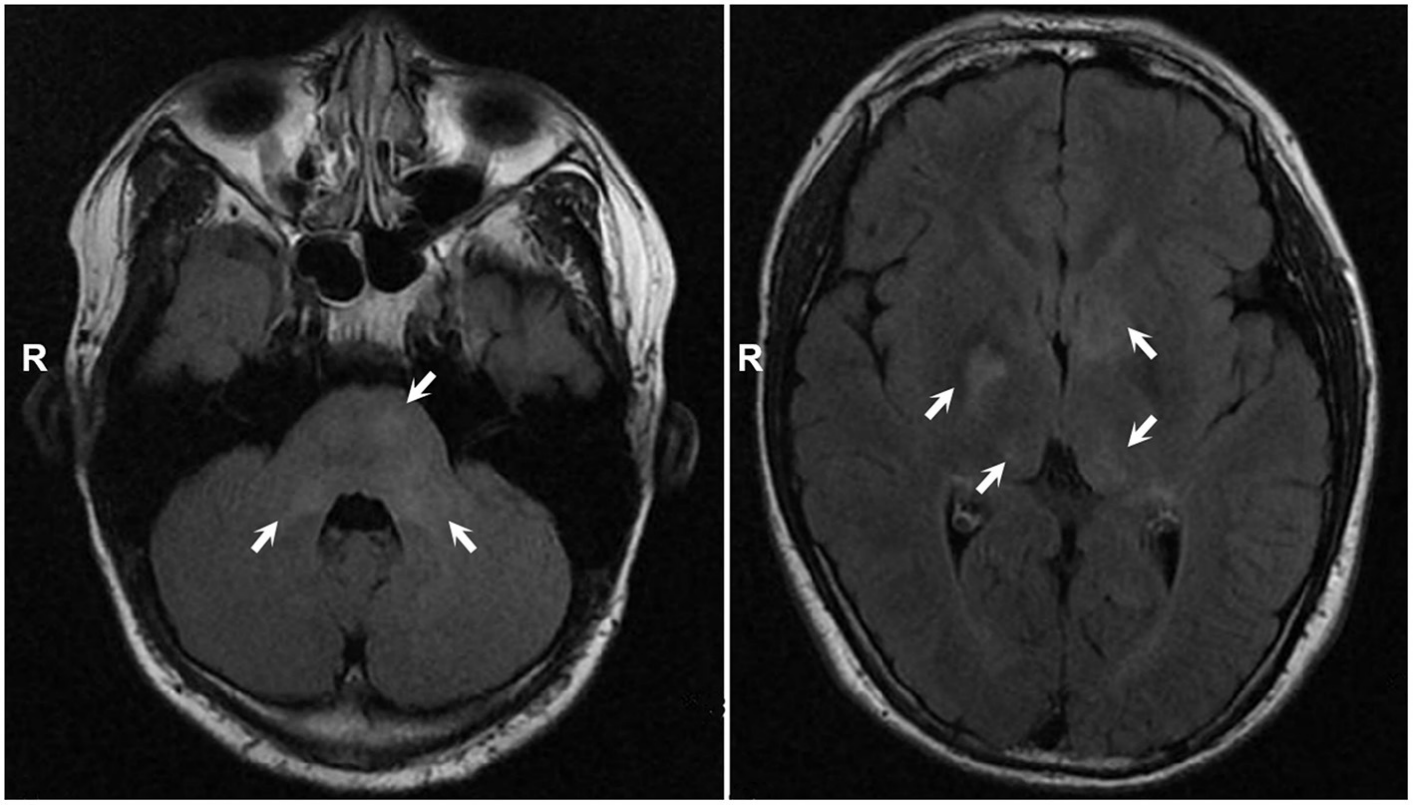
MRI showed multiple abnormal signal foci in bilateral basal ganglia, thalamus, and pons.

Systemic examination revealed a light brown plaque with clear boundaries of different sizes scattered on the chest and abdomen, back, shoulder, and left upper arm, not protruding from the skin surface, and with a diameter of 0.3–8.0 cm (Figure 1). The patient's mother had similar skin manifestations, but did not have headaches. The laboratory examination results were unremarkable. We performed a lumbar puncture and cerebrospinal fluid examination, which showed no significant abnormalities. Both autoimmune encephalopathy antibodies and central nervous system (CNS) demyelinating antibodies were negative. MRI of the head revealed multiple abnormal signal foci in the bilateral basal ganglia, thalamus, and pons (Figure 2). Based on these clinical and MRI findings, we suspected that the patient had NF1.

**Figure 3:**
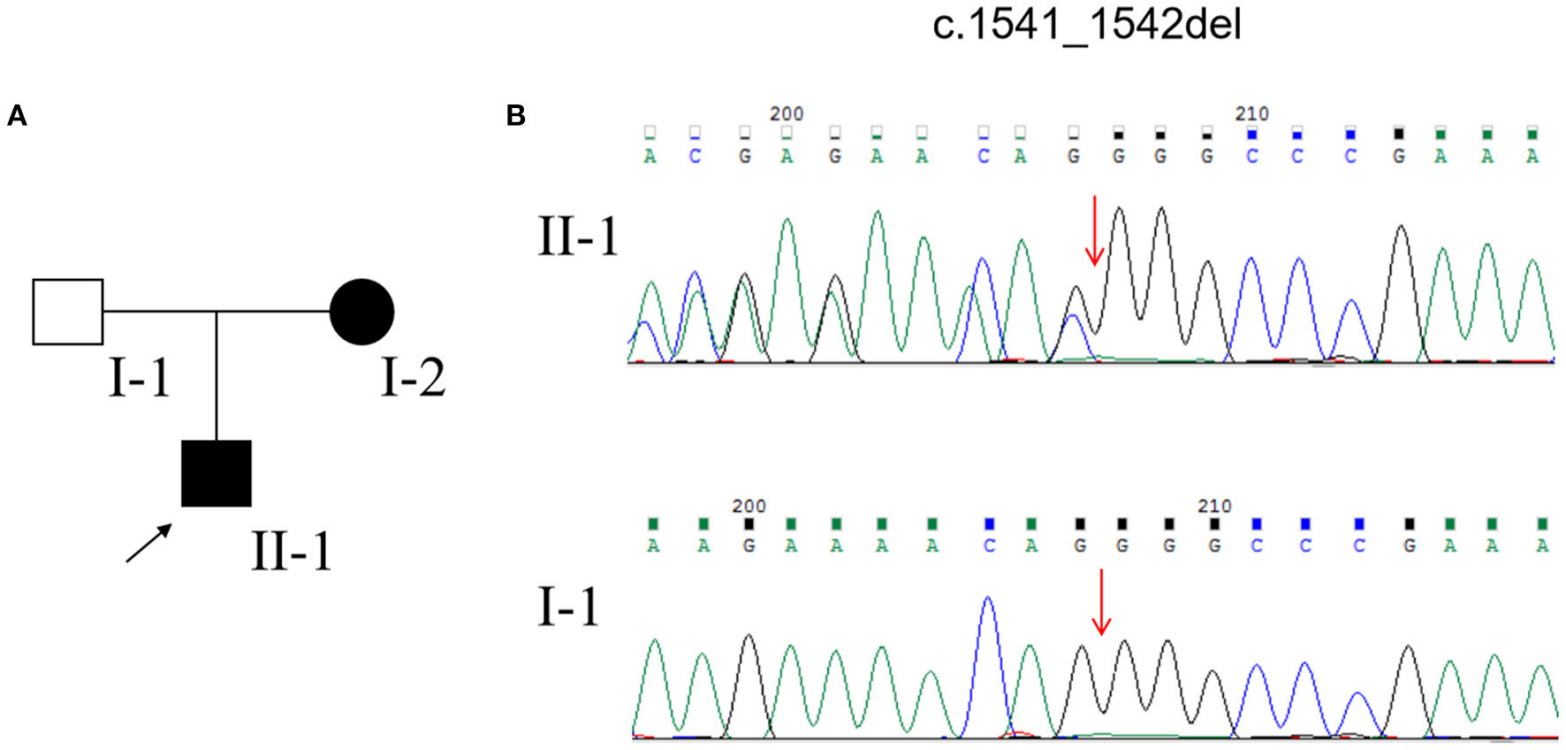
**(A)** Pedigree of the family affected with NF1. **(B)** Sequencing results of the NF1 mutation.

DNA extraction for WES and MLPA for *NF1* and *NF2* genes from the peripheral blood of the pedigree was performed after obtaining written informed consent. After data filtering, we identified a frameshift mutation (c.1541_1542del; p. Q514Rfs^*^) in the *NF1* gene in the proband and his mother (Figure 3). The same mutation was not detected in the proband's father (Figure 3). Based on the above clinical symptoms, MRI findings, and genetic testing results, the patient was diagnosed with NF1.

We then administered relevant symptomatic supportive treatment to the patient. After 4 months of follow-up, the frequency and severity of the patient's headache had reduced compared with those previously reported by the patient, and no progression of NF1-related complications was found. We will continue to follow-up the patient and adjust the treatment plan according to changes in his condition.

## Discussion

The patient had multiple skin café au lait spots and pigmentation and had a family history of similar conditions, but neither he nor his parents noticed these symptoms. Doctors at local hospitals were also ignored. The patient was transferred to our hospital with headache of unknown cause and was eventually diagnosed with NF1 by genetic testing.

NF1 is a syndrome characterized by a series of clinical symptoms caused by mutations in the *NF1* gene, which can affect multiple organ systems in the skin, nervous system, skeleton, and eyes (12). Although international diagnostic criteria for NF1 have been proposed, they do not include all clinical manifestations of NF1. Therefore, clinicians should not blindly comply with these diagnostic criteria to avoid missing atypical cases before obtaining genetic testing results. In addition to headaches, we have also summarized some atypical clinical symptoms of NF1 reported in previous English-language literature, which have not been included in the diagnostic criteria, to help enhance the vigilance of clinicians (Table 1).

**Table 1:** Atypical symptoms of NF1 cases reported in English clinical literature.

**Patient number**	**Atypical symptoms**	**Age**	**Sex**	**Familial history**	**NF1-related features**	**References**
1	Asthenia (case 1)	10	Male	Father and brother	Cafe-au-Lait spots	Julien et al. (7)
2	Asthenia (case 2)	14	Male	ND	Cafe-au-Lait spots Axillary freckles Plexiform neurofibromas Lisch nodules	Carman et al. (13)
3	Abdominal pain (case 1)	51	Female	ND	Cafe-au-Lait spots Axillary freckles	Adedayo et al. (5)
4	Abdominal pain (case 2)	49	Female	ND	ND	Lydia et al. (6)
5	Shortness of breath (case 1)	63	Female	ND	Cafe-au-Lait spots Cutaneous neurofibromas	Paul et al. (9)
6	Shortness of breath (case 2)	26	Female	Father and brother	Cafe-au-Lait spots Cutaneous neurofibromas	Alptekin et al. (14)
7	Neck and arm pain (case 1)	24	Male	ND	Cafe-au-Lait spots Intraspinal neurofibroma	Arvind et al. (15)
8	Neck and arm pain (case 3)	40	Female	ND	Mutiple neurofibromas	Arvind et al. (15)
9	Foot Pain	24	Male	ND	Axillary freckles An intraneural neurofibroma	Vasilis et al. (16)
10	Trigeminal pain	28	Female	Mother	Cafe-au-Lait spots Axillary freckles Cutaneous neurofibromas	Bianco et al. (17)
11	Overgrowth of left leg	52	Female	ND	Cafe-au-Lait spots Axillary freckles Cutaneous neurofibromas Lisch nodules	Tripolszki et al. (18)
12	Developmental retardation	10	Female	ND	Cafe-au-Lait spots	Havlovicova et al. (19)
13	Epilepsy	10	Female	ND	Cafe-au-Lait spots Axillary freckles Cutaneous neurofibromas Lisch nodules	Mastrangelo et al. (20)

ND, No data.

Headache is not uncommon in NF1 patients, but its cause is unclear. NF1 patients with headache were mostly caused by nonsense mutations, frame shift mutations and splicing mutations in the NF1 gene, which can lead to truncated protein and cause more serious clinical manifestations (21–24). Some other studies have suggested that the occurrence of headache in NF1 patients may be related to imaging abnormalities (mostly bright hippocampi and focal areas of signal intensity) (25). The lesions may purportedly affect the brain structures involved in headache pathophysiology, such as the brainstem and thalamus, and abnormalities in these regions have been reported in the scans of nine migraine patients (25, 26). We also observed multiple abnormal signal foci in our patient's thalamus. This suggests that the patient's headache might have been related to these imaging abnormalities. However, in addition to NF1, other CNS diseases, such as infection, autoimmune encephalopathy, and CNS demyelination, may also lead to similar imaging findings. Therefore, we performed lumbar puncture and cerebrospinal fluid examination, including assessments for autoimmune encephalopathy antibodies and CNS demyelinating antibodies, all of which yielded negative results. This suggests that the patient's imaging abnormalities were most likely due to NF1 rather than other diseases. However, some studies suggest that although NF1 patients have intracranial structural changes or an increased frequency of cerebral anomalies, this is not a causal relationship with headache (27–29). Some patients also develop headache without intracranial lesions. Therefore, further studies are needed to investigate the pathophysiological mechanisms of headache in NF1 patients.

NF1 is the result of mutations in the tumor suppressor gene *NF1* on chromosome 17q11.2, and more than 2,600 different *NF1* gene mutations have been found in the Human Gene Mutation Database (30). *NF1* mutations are diverse, including missense/nonsense, splicing, microdeletion, microinsertion, total deletion, total insertion, and complex rearrangement (12). Therefore, gene detection should include WES and MLPA to avoid omission. Consequently, NF1 patients are many times more likely to develop tumors that are most common in the nervous system, such as optic gliomas, neurofibromas, and malignant peripheral nerve sheath tumors (31). Tumors of the skin and gastrointestinal tract are also common (32). This may be an important reason why the lifespan of NF1 patients is 15 years shorter than that of the general population (33). Therefore, it is particularly important to conduct genetic testing for patients with suspected NF1 as soon as possible. This is conducive to the early detection, diagnosis, and treatment of NF1 to improve prognosis. However, the clinical manifestations and severity of NF1 vary widely between members of the same family and between two individuals with the same mutation. Previously, GIOVANNI reported a 29-year-old Italian woman with giant elephantiasis (34). The mutation was consistent with our reported case after genetic testing (c.1541_1542del; p. Q514Rfs^*^); thus, the patient was diagnosed with NF1 (35). However, the severity and symptoms of the two diseases are quite different. The female presented mainly with giant elephantiasis of the right leg, which was histopathologically identified as a lymphangioma, with multiple neurofibromas and few café au lait patches. The patient did not exhibit any symptoms of headache nor did he have relevant head MRI findings similar to our case. The reasons for this large difference in phenotypes are complex and may be related to differences in race, environment, and lifestyle. This suggests that, although genetic testing can play a role in the early detection and diagnosis of NF1, clinicians still need to make personalized treatment plans for patients according to their different clinical manifestations.

The current treatments for NF1 mainly focus on early detection, symptomatic treatment, and treatment of complications (36). The patient had no typical symptoms or complications other than cutaneous manifestations. Therefore, in addition to the symptomatic treatment of headache, he will also need long-term follow-up, timely detection, and treatment of related complications.

## Conclusion

In this article, we have reported the case of a 13-year-old Chinese NF1 patient with prominent headache. WES and MLPA revealed a frameshift mutation. We have also reviewed the atypical clinical manifestations of NF1 patients reported in English in the past to improve the alertness of clinicians and to emphasize the importance of early genetic testing for these patients.

## Data availability statement

The datasets presented in this article are not readily available because of ethical and privacy restrictions. Requests to access the datasets should be directed to the corresponding author/s.

## Ethics statement

The studies involving human participants were reviewed and approved by the Ethics Committee of Xiangya Hospital, Central South University. Written informed consent to participate in this study was provided by the participants' legal guardian/next of kin. Written informed consent was obtained from the individual(s), and minor(s)' legal guardian/next of kin, for the publication of any potentially identifiable images or data included in this article.

## Author contributions

MG and HL conceptualized the study and drafted the initial manuscript. MG, HL, QS, and GY analyzed and explained the data. QS have collected the data. MG, HL, and GY conceptualized and designed the study, and critically reviewed and revised the manuscript. All authors contributed to the article and approved the final version of the manuscript.

## Funding

This work was supported by grants from the Natural Science Foundation of Hunan Province, China (2021JJ41045, 2021JJ31093) and Natural Science Foundation of Changsha, China (kq2014279).

## Conflict of interest

The authors declare that the research was conducted in the absence of any commercial or financial relationships that could be construed as a potential conflict of interest.

## Publisher's note

All claims expressed in this article are solely those of the authors and do not necessarily represent those of their affiliated organizations, or those of the publisher, the editors and the reviewers. Any product that may be evaluated in this article, or claim that may be made by its manufacturer, is not guaranteed or endorsed by the publisher.
